# A six-year teaching life supportive first aid program to eventually generate peer trainer pupils: a prospective case control study

**DOI:** 10.1186/s12909-023-04476-x

**Published:** 2023-07-05

**Authors:** Berndt von Amelunxen, Samantha Kirk, Julian Hind, Jennifer Illibauer, Christoph Krall, Sebastian Lessing, Aurelien Noyelle, Peter M J Murphy, Fritz Sterz

**Affiliations:** 1 Department of Emergency Medicine, Medical University of Vienna, Wien, Austria; 2 Center of Medical Statistics, Medical University of Vienna, Wien, Austria; 3 Vienna International School, Wien, Austria; 4 Allgemeines Krankenhaus Wien Medizinische Universität Wien, Universitätsklinik für Notfallmedizin, Währinger Gürtel 18-20/6D, Wien, 1090 Austria

**Keywords:** Cardiopulmonary resuscitation, Learning, Schools, Teaching

## Abstract

**Background:**

Out of hospital cardiac arrest is a life-threatening condition. To improve the chances of survival, lay-person cardio-pulmonary-resuscitation (CPR) is a crucial factor. Many bystanders fail to react appropriately, even if life supporting first aid (LSFA) programs and campaigns including CPR tried to increase the handling of basic cardiac life support. To achieve an enhanced learning of CPR a pupil’s grade after grade teaching program was established in a school with medical students.

**Methods:**

The learning of CPR was investigated in a prospective, case-controlled study at an international school. Pupils (12 ± 3 years old) joining our LSFA courses (n = 538, female: 243, attendance for evaluation: 476) were compared to a control group (n = 129, female: 52, attendance for evaluation: 102). Surveys and quality of CPR (QCPR%) through a computer linked “Resusci Anne” dummy were compared with Chi-squared tests, t-tests pair wisely, and by one-way ANOVA.

**Results:**

Knowledge and skills on the “Resusci Anne” were significantly better in trained grade 9 pupils compared to the control group (QCPR, 59 vs. 25%). The number of LSFA courses each grade 9 student had, correlated with improved practical performance (r^2^ = 0.21, p < 0.001). The willingness to deliver CPR to strangers increased with improved practical performance. Attitudes towards performing CPR were high in all participating grades.

**Conclusion:**

Repetitive teaching LSFA to grade 5–9 pupil’s grade after grade by medical students has been successfully established. Pupils who finish the program will eventually be able to teach LSFA to younger students. This is furthermore a good way of sharing a “learning by teaching” role and it enables to have more pupils as trainers who can provide instruction to a larger number of pupils with the purpose of having a better-trained population in LSFA.

## Background

The importance of bystander cardiopulmonary resuscitation (CPR) is well known [1, 2]. The efforts to increase public awareness about the consequence of bystander CPR have been high. Little progress has been made. As a result the survival rates of cardiac arrests have remained much lower than what they could be [3]. Most citizens, even those who received first aid training, do not feel confident and/or perform CPR correctly in more than 50% cases of an emergency [4, 5]. The lack of response is mainly due to deficiencies in life supporting first aid (LSFA) education including CPR [6, 7]. Even programs in schools are often hard to integrate into the school-system, when depending on teachers or medical professionals to run the LSFA courses. In Denmark, for example, it has been shown how difficult it is to implement CPR training in schools [8].

The original program of “teaching LSFA by medical students in schools” is an example of how LSFA could be taught to a city or country’s broader population [6, 7, 9, 10]. It is based on the hypothesis of learning opportunities for medical students in the teacher’s role via altering their cognitive processes and fostering higher motivation for LSFA through interactive and practical teaching as already used for other interventions in medical education [7, 11–15]. Unfortunately, this version of the “teaching by medical students program” also faces a staffing limitation. Even if all medical students joined this program, they would not be enough to teach LSFA at all schools. Another limitation of this program is that pupils were only once taught a single course of LSFA. A recent study shows that groups with more than one LSFA course show greater confidence in their skills and performance of higher quality CPR (QCPR) [16]. Due to the limitations of the current “teaching program” a new method of recruiting more potential trainers had to be found.

We developed a modified LSFA training system to the original program using initially medical students as trainers for pupils [6]. To achieve an improved learning of CPR repetitive teaching LSFA to grade 5–9 pupil’s grade after grade has been established. Therefore, we compare (1) grade 9 students (who received the intervention for six years) to grade 10 students (who did not receive the intervention), in order to know if the intervention is effective, and (2) grades 5 to 9, who have been involved in the intervention for a different period of time, to understand how learning unfolds throughout the years of intervention. The aim is to observe whether the pupils who graduate from this program have better theoretical and practical knowledge than those who did not. It will be the basis for further developments, if older pupils from this program can pass on their knowledge by training the younger ones so that LSFA training in schools is not limited by a lack of trainers and thereby also comply with the ambition to enhance “learning by teaching LSFA”.

## Methods

This prospective case control study took place at the Vienna International School (VIS) in cooperation with the Medical University of Vienna (MUV).

### Pupils

Medical students taught voluntarily recruited pupils LSFA for the first time during the first semester of their fifth grade, during a double period of 80 min physical education (PE). Every year the following fifth grade started the same program and it was repeated every year, grade after grade, once per semester from grade 5 to 9. The medical students acquired the necessary qualifications through a special training at the MUV.

The first lesson at the VIS included a presentation, a game called “Stuck in the first aid”, ~ 15 min of CPR practice on LittleAnne & MiniAnne dummies (https://laerdal.com/de/products/simulation-training/resuscitation-training/) and finding and using an automatic external defibrillator (AED). During the second semester of the school year a recap was held (5 months after the initial course). The pupils were asked questions regarding what they remembered and discussed as key elements were emphasised. All the upcoming recaps over the following four years were adjusted to last no longer than 40 min each during PE lessons. Since it is an international school, it is not uncommon for some pupils to leave the school and for new ones to join at any time. The pupils with more experience were included in the process of explaining newly enrolled pupils what to do. In grade seven they learned the “10 seconds check” and were practicing their first “real life scenario” on a dummy. Real defibrillators on the school campus were swapped to practice defibrillators for the scenario and the pupils could use any utilities in the school that they found necessary to save a life. This included calling for help, getting a teacher and telling the security at the front entrance that the ambulance will arrive.

In the second semester of grade nine and final course of the program there was a free practice lesson. Many pupils used this lesson to prepare and practise for the exam, which pupils can take after finishing this program. Whoever passed the exam got a certificate from the VIS and MUV. With this certificate they received the allowance to join a training at the MUV, where they learned how to teach LSFA themselves. The plan was to use such pupils with their required competence, to teach LSFA to younger pupils at the VIS. As an additional motivation for teaching LSFA the pupils were able to do this as part of a mandatory community service program of the school and add a boost to their CV’s.

### Evaluation

The evaluation took place at the end of the 2017 summer semester for all grades, five years after initiation of the program.

The pupils’ theoretical knowledge of all grades was assessed with a questionnaire in which they had to answer 11 questions (listed as adjunct to the end of the manuscript) during all LSFA courses. To show the individual progress of the pupils of all grades five to nine were evaluated with assessment sheets and a so called “Resusci Anne®” (Laerdal Medical GmbH, Vienna, Austria) dummy, which is linked to a computer and measures the quality of CPR (= QCPR%). After two minutes a percentage is calculated by measured speed and strength of compressions, hands-off time, and the effectiveness of ventilations. A cut-off value of 60% was set to indicate being able to perform CPR. Reasons why pupils did not achieve 60% were that the compressions were not deep enough; they compressed in the wrong place; used a rhythm very different to 30:2; had long hands-off times; completely failed ventilations; or a combination of these. Everything beyond 80% indicated a very good performance.

### Data analysis

Quantitative variables are reported by mean and standard deviation, qualitative variables are reported by percentage. A score for theoretical knowledge was calculated as sum of correct answers to questions about steps of first aid, correct circle and speed of CPR and usage of the defibrillator. Score points between groups were reported by mean and standard deviation and compared between groups by t-test pair wisely and by a one-way ANOVA with post hoc Tukey-tests for more than two groups. Answers to theoretical questions were trichotomized into “all answers correct”, “partly correct” and “not correct”, reported as percentages of completely correct answers and compared between groups by a Chi-squared test. Grades 5 to 9, who have been involved in the intervention for 5 years were evaluated and grade 9 students to grade 10 students (control group) that did not participate in this program were compared. Half of the grade 10 pupils had the option to participate in a first aid training needed for the driving license. That allowed us to further compare the pupils of the program to this ‘special’ subgroup of the control group. Out of 102 pupils of the control group 44 never had any teaching in LSFA and 46 pupils participated in the first aid training for the driving license, and 12 pupils had some kind of LSFA training. Logistic regression models for each grade between the willingness to deliver CPR increasing practical performance will be plotted.

## Results

### Pupils

The number of pupils joining the LSFA courses was 538 [grade 5: 86, grade 6: 109, grade 7: 111, grade 8: 110, and grade 9: 122] with a total attendance for evaluation of 476 (88%) [grade 5: 78 (91%), grade 6: 96 (88%), grade 7: 101 (91%), grade 8: 95 (86%), and grade 9: 106 (87%)]. Their mean age was 12 ± 3 years and female were 243 (52%). The number of pupils in the control group was 129 with an attendance of 102 (79%); females were 52 (51%). In grade 9 the pupils participated on average 6.3 courses out of the 10 offered (range: 0–10; with 38 pupils participating in all 10 units of the program). The number of pupils in the control group with prior participation in first aid instructions required for the driving licence was 46 (45%). The number of pupils in the control group with previous knowledge from trainings, other than the one required for the driving licence, that had received prior CPR training was 12 (12%).

### Evaluation

The theoretical knowledge showed a better performance in the pupils participating in this program compared to the control group. A t-test identified higher performance of grade 9 than of control pupils (p < 0.001). Results were almost identical when grade 9 pupils (attending all training units) and only control pupils participating in the first aid instructions necessary for a driving license were compared (p < 0.001). No differences were identified between pupils of grade 5–9 by a one-way ANOVA (p = 0.245). Grade 9 pupils fared significantly better than control pupils when comparing percentages of completely correct answers about steps of first aid (p < 0.001), speed of CPR (p < 0.001) and application of the defibrillator (p = 0.01). No difference between percentages of completely correct answers between grades 5 to 9 can be identified by a chi-squared test, with percentages ranging between 55% in grade 9 and 62% in grade 5. For correct circle and rhythm of CPR there were no full achievers among the pupils. For correct circle percentages of partial achievers ranged from 49% (grade 6) to 73% (grade 5). Chi-squared tests showed differences in proportions of partial achievers between grade 6 and grade 5 (p = 0.004) and between grade 7 and grade 5 (p = 0.028). For rhythm, percentages of partial achievers increased continuously from 53% (grade 5) to 90% (grade 9). Pairwise chi-squared tests showed differences in proportions of partial achievers between grade 5 and grades 7–9 (p = 0.028. 0.03, < 0.001 resp), between grade 6 and grades 8–9 (p = 0.031, 0.035, < 0.001 resp), between grade 7 and grade 9 (p = 0.004) and between grade 8 and grade 9 (p = 0.004). Comparing pupils from the training program with the control group for rhythm of CPR, a chi-squared test identified a higher percentage of partial achievers in grade 9 (90%) than in control (67%) pupils (p < 0.001), whereas no substantial difference with respect to correct circle between grade 9 (40%) and control (35%) pupils could be observed (p = 0.61). The most common mistake was believing that the defibrillator would always give a shock. Looking at this question, the only grade with significant difference between pupils who attended the maximum number of courses and the rest of the class was grade 5 (50% vs. 17%, p = 0.004). A t-test identified higher confidence in grade 9 (6.2 ± 2.3) than in control group (4.1 ± 2.7) pupils, but no difference in confidence between grade 5 to 9 pupils could be identified by a one-way ANOVA (p = 0.09).

**Fig. 1:**
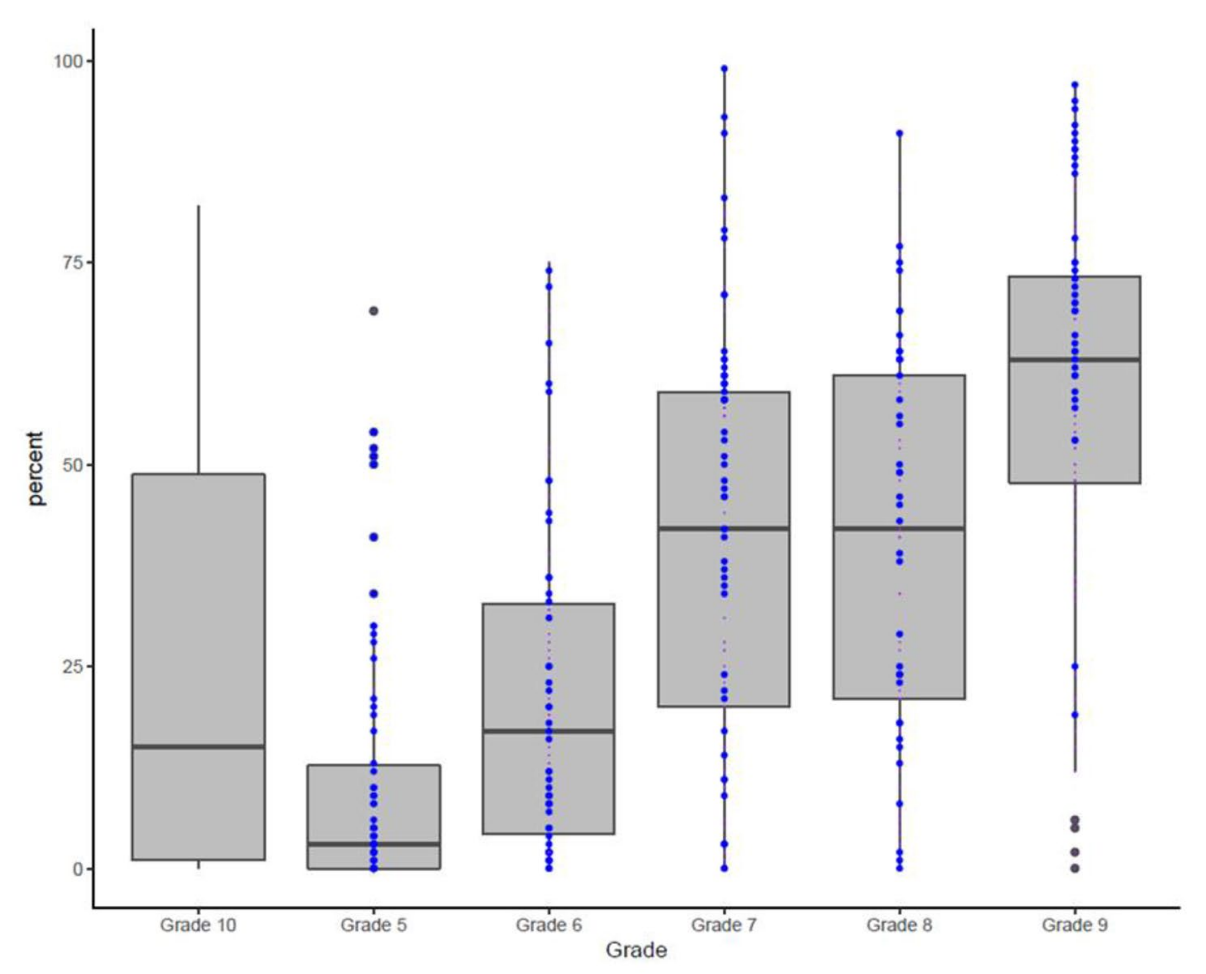
Percentages reached on the Resusci Anne per grade and control group. The blue dots indicate the pupils who attended the maximum number of courses

The best QCPR percentages reached on the “Resusci Anne” were achieved by grade 9 pupils. A t-test identified higher performance of grade 9 pupils than in control pupils as measured by QCPR% (p < 0.001). The difference was even more striking when grade 9 pupils (attending all our LSFA courses) and only control pupils participating in the first aid instructions necessary for a driving license were compared (p < 0.001). A positive correlation between number of LSFA courses and QCPR% could be observed in grade 7 (r^2^ = 0.07, p = 0.01) and in grade 9 pupils (r^2^ = 0.21, p < 0.001). A one-way ANOVA with post-hoc Tukey tests identified differences between grade 5–9 in QCPR% with p < 0.001 except for the difference between grade 6 and grade 5 (p = 0.005) and between grade 8 and grade 7 (p = 1). A continuous increase from grade 5 (10 ± 15) to grade 9 (59 ± 23) pupils can be observed.(Fig. 1).

**Fig. 2:**
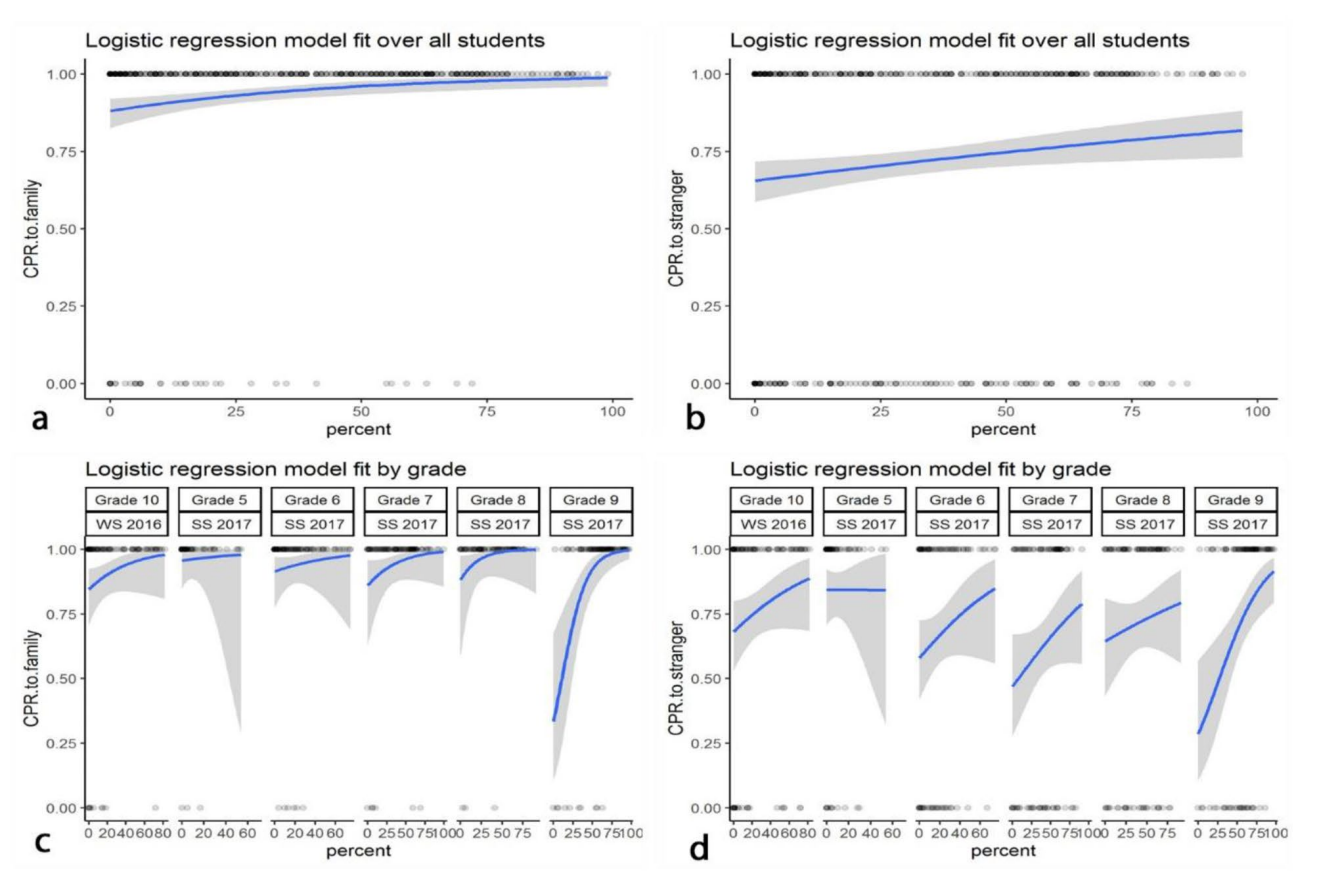
Logistic regression models that compare the practical performance of the pupils on the Resusci Anne with the readiness to perform life-supporting first aid to strangers **(a)** and family **(c)**. Logistic regression models are plotted for each grade **(b** & **d**, respectively)

Logistic regression identified an increase of willingness to deliver CPR to a stranger with increasing practical performance (est: 0.002, 95% C.I.:[0.010,0.016], p = 0.004). (Fig. 2a). Plotting logistic regression models for each grade shows that grade 9 student’s decision to deliver CPR to strangers is much more dependent on their actual performance than in lower grades and in the control group. (Fig. 2b) The dependence on performance (est: 0.02, 95% C.I.[0,01, 0.04], p = 0.007) and the same trend among grades can be seen in the student’s readiness to deliver CPR to family members. (Fig. 2c, d)

## Discussion

Teaching LSFA with medical students to generate peer teacher pupils for a school based self-running system has shown to be an effective method in educating young pupils about CPR and in their self-confidence about LSFA.

Our data compared to previous studies have demonstrated that school pupils can retain knowledge regarding this matter even better than adults in other trainings and that young children are easier to motivate towards this topic [9, 17, 18]. Our educational strategy is additionally supported by others showing increased pupils’ acquisition, retention, promotion and dissemination of resuscitation techniques [9, 10, 19, 20].

The degree of confidence and security in the performance and QCPR% was related to the yearly retrieval practical classes with the use of mannequins as similarly shown with other learning strategies [21]. A positive correlation was found between the number of trainings attended and the theoretical knowledge about LSFA and QCPR% achieved. (Fig. 1) In countries such as New Zealand standard one time first aid courses were investigated and concluded that with the current standard first aid teaching styles it is not likely for pupils to perform adequate CPR [22, 23] While arguing that more lessons result in a better performance, the body size and weight is an additional factor which can affect the results [24], [25], [26, 9, 17, 18]. The results from the analysis of the student’s attitude to perform CPR to strangers and family members regardless of their performance level is also in tune with the finding of others [5]. The high number of CPR recaps that aid the pupils in solidifying these skills ultimately leaded to higher self-confidence as already shown by Lukas et al. [27].

In addition, after six years and a total of 10 completed courses the most advanced pupils who passed an exam taught the younger pupils. In the first year of pupils teaching pupils 10 pupils signed up (female, 40%). In the second year 23 pupils signed up (similar quote). Data on these following years in which former pupils teach younger students will be provided for the peer instructors and the younger students in upcoming analysis.

Future lines of research, hypothesizing that the project could become a learning by teaching intervention will also analyse the learning of medical students and of former pupils taking on the role as teachers.

Unfortunately, a downfall of this study is that the school has a high dropout quote since it is an international school, and many pupils only stay for 1 or 2 years. The VIS is a big school though and the number of pupils in each grade was around 100. Even though the dropout quote was high, the absolute numbers still represent a good amount of data, which supports the benefits of expanding this program.

## Conclusion

The findings of this pilot program demonstrate that incorporating LSFA lessons in schools is a plausible solution to increasing the odds of having more bystanders in the population that are willing to help in the case of an emergency. It also serves as a working model to show how pupils can be incorporated in training other pupils to perform LSFA. However, whether this program will affect the overall survival is still to be determined. Further research, after implementing this program in multiple schools to cover a bigger area, will have to evaluate this topic and to focus on the learning of students in the role of teachers (both medical students and former pupils who are now teaching younger students) for making the program sustainable in the long-term. The implications of the findings of this study employing novel teaching practices on global medical or health professions education could be relevant to improve the current practices on teaching LSFA. The Medical University with students teaching basic life support to school children as a required element of medical education, going beyond its walls and interacting with the community, will play an important part in social accountability.

## Data Availability

Datasets, if not presented in the main manuscript are available via the first author Bernd von Amelunxen and/or statistician Christoph Krall of the manuscript.
